# Obtaining baselines for eating disorder patients in hospital settings: does time to assessment matter?

**DOI:** 10.1186/2050-2974-2-S1-O47

**Published:** 2014-11-24

**Authors:** Eva Vall, Tracey Wade

**Affiliations:** 1 School of Psychology, Flinders University, Adelaide, Australia

## 

Capturing reliable baseline information from patients receiving treatment for an eating disorder (ED) is critical for researchers and clinicians. Inaccurate information has implications for the interpretation of subsequent measurements. In naturalistic environments such as busy hospital wards, operational demands can interfere with data collection processes, and time taken to conduct baseline assessments may vary. The current study examined whether this variation impacted on baseline measurements in an inpatient setting. We assessed adult (n = 35) and paediatric (n = 28) patients at varying lengths of time after admission, ranging from 0 to 7 days (m = 1.86, SD = 1.49). Time was dichotomised into early assessment (within 24 hours) and late assessment (post 24 hours). For paediatric but not adult patients, time of assessment predicted scores on motivation to recover and ED psychopathology, both before and after controlling for admission BMI, with moderate to strong effect sizes. Lower motivation and higher ED pathology were observed in the early assessment group, compared to the later group. Our findings raise an important consideration for the assessment of paediatric ED inpatients. A stringent time protocol should be applied to all admission assessments, or time of assessment effects should be considered in data analyses.

This abstract was presented in the **Assessment** stream of the 2014 ANZAED Conference.

